# An approach to consider behavioral plasticity as a source of uncertainty when forecasting species' response to climate change

**DOI:** 10.1002/ece3.1519

**Published:** 2015-05-25

**Authors:** Antonio-Román Muñoz, Ana Luz Márquez, Raimundo Real

**Affiliations:** 1 Biogeography, Diversity and Conservation Research Team, Department of Animal Biology, Faculty of Sciences, University of MalagaE-29071, Malaga, Spain; 2 Department of Didactic of Science, Faculty of Science Education, University of MalagaE-29071, Malaga, Spain

**Keywords:** *Aquila fasciata*, behavioral plasticity, climate change, Iberian Peninsula, modeling updating, species distribution models

## Abstract

The rapid ecological shifts that are occurring due to climate change present major challenges for managers and policymakers and, therefore, are one of the main concerns for environmental modelers and evolutionary biologists. Species distribution models (SDM) are appropriate tools for assessing the relationship between species distribution and environmental conditions, so being customarily used to forecast the biogeographical response of species to climate change. A serious limitation of species distribution models when forecasting the effects of climate change is that they normally assume that species behavior and climatic tolerances will remain constant through time. In this study, we propose a new methodology, based on fuzzy logic, useful for incorporating the potential capacity of species to adapt to new conditions into species distribution models. Our results demonstrate that it is possible to include different behavioral responses of species when predicting the effects of climate change on species distribution. Favorability models offered in this study show two extremes: one considering that the species will not modify its present behavior, and another assuming that the species will take full advantage of the possibilities offered by an increase in environmental favorability. This methodology may mean a more realistic approach to the assessment of the consequences of global change on species' distribution and conservation. Overlooking the potential of species' phenotypical plasticity may under- or overestimate the predicted response of species to changes in environmental drivers and its effects on species distribution. Using this approach, we could reinforce the science behind conservation planning in the current situation of rapid climate change.

## Introduction

Human activities are causing major environmental modification, including habitat destruction, fragmentation, and degradation, and, as a result, many populations are exposed to novel perturbations and declines are occurring all over the world (Scheffer et al. 2001). On the other hand, these modifications may constitute new opportunities for species to take advantage of both ecologically and evolutionarily (Reid et al. 2007). New selective pressures on phenotypic traits may arise from the interaction of individuals with their new, modified local environment, which consists of abiotic and biotic factors. Phenotypic traits, including individual behavior, may respond to these pressures under the constraints imposed by the organism's genetic architecture, and this response in turn affects how individuals shape their environment (Ferrière et al. 2004). This causal relationship, from the environment to the individuals, and back, defines the environment feedback loop that intimately links ecological and evolutionary processes.

Over the past decades, human-intensified climatic change is becoming an additional pressure on natural populations (Loarie et al. 2009). The 2013 Report of the Intergovernmental Panel on Climate Change (IPCC 2013) confirms that warming in the climate system is unequivocal, with many of the observed changes unprecedented over decades to millennia and likely without precedent during the last 10,000 years, according to palaeoclimate data (IPCC 2001). Species could respond to the effects of global warming, among others, by shifting their geographical distribution (Parmesan and Yohe 2003; Barbet-Massin et al. 2009), changing the timing of growth and reproduction (Franks et al. 2007; Pulido and Berthold 2010), and also undergoing evolutionary adaptations (Williams et al. 2008).

There is recent evidence that evolutionary changes driven and constrained by ecological interactions can be rapid (Bradshaw and Holzapfel 2006; Parmesan 2006; Hoffmann and Sgro 2011; Ozgo 2011). These evolutionary changes may be critical for the resilience of ecosystems challenged by environmental modifications on a wide range of temporal and spatial scales. This point out that evolutionary adaptation could be an important way for natural populations to counter and adapt to rapid climate change. Hendry and Kinnison (1999) concluded that rapid microevolution could represent the norm rather than the exception in contemporary populations confronted with environmental change. Adaptive changes could enable species for exploiting newly favorable opportunities provided by a changing climate, enabling them to shift and expand their geographic ranges by the progressive establishment of new local populations.

In recent times, predictive modeling of species distribution has become an important tool in studies of ecology, biogeography, evolution, conservation biology, and climate change research (e.g., Guisan and Thuiller 2005). Models estimate the species' ecological requirements associating geographic distributions with sets of predictor variables, and are informative when investigating the possibility that particular changes in climate might affect distributions. As behavioral plasticity to environmental changes can be fast and strong, it should not be ignored when predicting the effects of climate change on species distributions. This point would help to produce better, more scientifically sound, forecasts of the effects of global warming on biodiversity. However, with some exceptions (e.g., Kearney et al. 2009; Sinervo et al. 2010), the importance of species' behavioral plasticity is normally ignored in predicted distribution shifts, colonization patterns, and species' responses to climate change.

An explanatory model was proposed for the distribution of an endangered species in Spain, the Bonelli's eagle *Aquila fasciata*, based on climate and topography (Muñoz et al. 2005). Subsequently, climate change was predicted to affect the distribution of this species throughout this century, favoring its expansion in Europe, although its cliff-nesting breeding habits may hinder its actual capacity to colonize new climatically favorable areas (Muñoz et al. 2013). Although the species normally breeds on cliff ledges, it shows some potential for adaptation to nest in other substrates as trees.

In this study, we take Bonelli's eagle as case study to propose a methodology based on fuzzy logic to model the future distribution of species taking into account their behavioral plasticity and capability to respond to the opportunities and challenges represented by different climate change scenarios.

## Materials and Methods

### Study area

**Figure 1:**
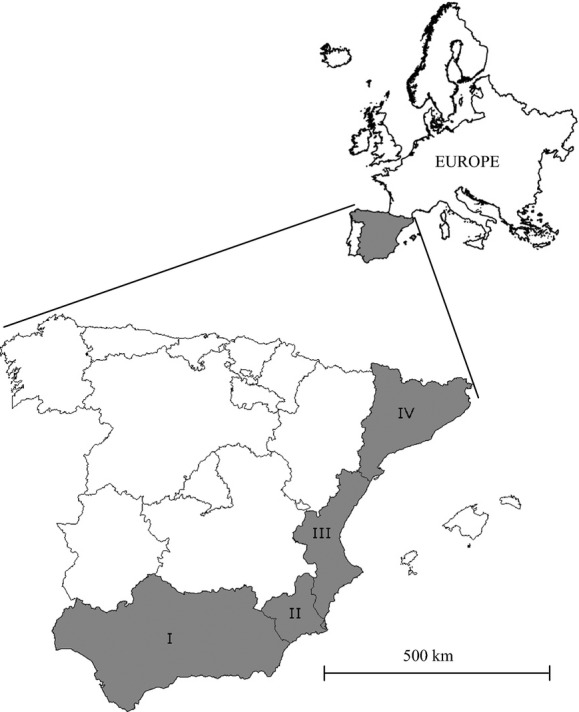
Study area, mainland Spain, with details of Mediterranean regions. I: Andalusia, II: Murcia, III: Valencia, and IV: Catalonia. The models' performance was assessed outside and inside the Spanish Mediterranean area.

**Figure 2:**
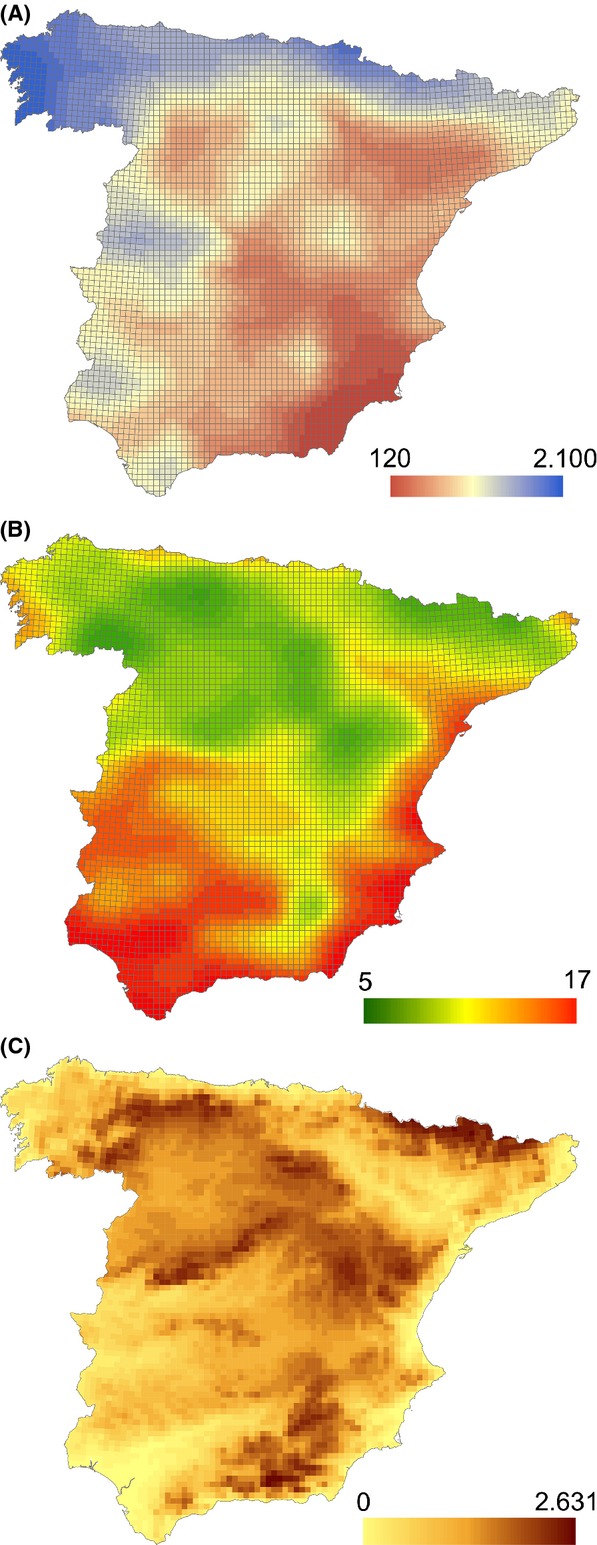
Distribution of mean annual precipitation (A), mean annual temperature (B), and mean altitude (C) in the study area.

The study area comprises the mainland Spanish territory, an area of 493,518 km^2^ characterized by an heterogeneous climate and located between the temperate climate of central Europe and the arid climate of northern Africa, which makes it particularly appropriate for analyzing different climate change scenarios (Fig.1) (Nogués-Bravo et al. 2008). There is a mainly eastward- and southward-decreasing gradient of precipitation and a mainly northward-decreasing gradient of temperature (Font 2000). Annual precipitation varies from less than 200 mm to more than 2000 mm, whereas mean annual temperatures vary from less than 6°C to more than 17°C (see Fig.2A and B, respectively). Mainland Spain has important mountain ranges, with a maximum altitude of 3478 m, many of them in coastal areas (see Fig.2C).

### Study species

We focused our study on Bonelli's eagle*,* a long-lived territorial raptor that suffered a severe population decline of 20–50% over the last decades in Europe (Rocamora 1994; Real 2003). The current estimated European population consists of 920–1100 pairs (BirdLife International 2004), of which approximately 80% are concentrated in our study area. On a global scale, it occupies mountains, cliffs, crags, gorges, hills, and plains with forest or woodland (Cramp and Simmons 1980), although in some areas, it may build its nest on lofty trees, as in southern India and Portugal (Ali and Ripley 2001; Palma 2010). In Spain, Bonelli's eagle is primarily a cliff-nesting species, with 95.5% of the nests found in this substrate, while trees and power lines are used in a small proportion, 4% and 0.5%, respectively (Del Moral 2006). Interestingly, in the domain of the Iberian Peninsula, in Portugal, the proportion of pairs nesting on trees is different, with 64% of the Portuguese population nesting in large eucalyptus, pines, and cork oaks (Palma 2010). Although in northern Portugal this species normally breed on cliffs, in southern Portugal, 94% of breeding pairs are tree-nesters (Palma 2010). This makes obvious the plasticity of the species when choosing nesting substrate, being able to breed in trees in those favorable areas where mountains are scarce.

### Target and predictor variables

**Figure 3:**
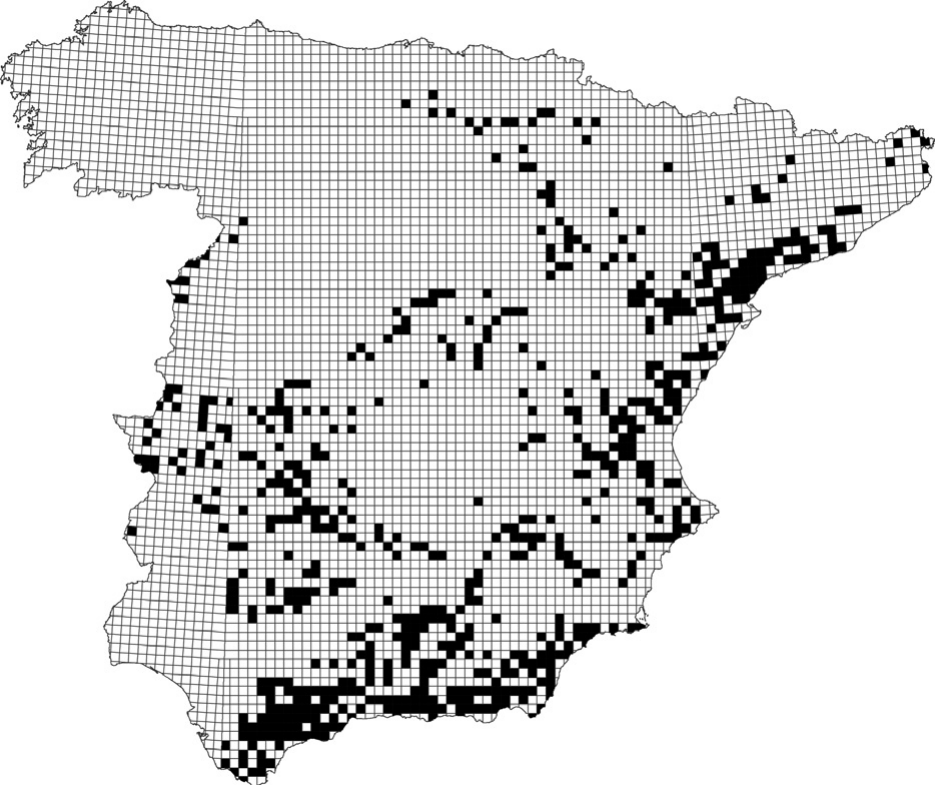
Present Bonelli's eagle distribution based on UTM 10 × 10 km squares in mainland Spain. Black squares represent breeding territories presences (data taken from Del Moral 2006).

To model the species' distribution, we used the data provided by the last national survey, carried out in 2005 (Del Moral 2006) (see Fig.3). The fundamental unit for the species distribution in mainland Spain was the UTM 10 × 10 km square, with a total of 5167 quadrats in the study area. We used 18 variables related to climate and topography (Table1). As highlighted by Rapacciuolo et al. (2014), we considered various aspects of climate to obtain range shifts more complex than expected solely from temperature changes. These factors were selected because topography and Mediterranean climate are consistent factors influencing the distribution of the species in Spain at different spatial scales; from a national scale (Muñoz et al. 2005; Carrascal and Seoane 2009), to more local scales centered in the near vicinity of the nesting sites (Gil-Sánchez et al. 2004; López-López et al. 2006; Muñoz and Real 2013).

**Table 1:** Variables used to model the species distribution grouped in explanatory factors and their sources

Factor	Variables	Code
Topography	Mean altitude^(1)^	*A*
	Second-order polynomial of Altitude	*A*^*2*^
	Slope (°) (calculated from altitude)	*S*
	Second-order polynomial of Slope	*S*^*2*^
	Southward exposure degree(°)^(2)^	*OrS*
	Westward exposure degree(°)^(2)^	*OrW*
Climate	Mean annual precipitation (mm)^(3)^	*PAn*
	Mean spring precipitation (mm)^(3)^	*PSp*
	Mean summer precipitation (mm)^(3)^	*PSu*
	Mean autumn precipitation (mm)^(3)^	*PAu*
	Mean winter precipitation (mm)^(3)^	*PWi*
	Mean annual temperature (°C)^(3)^	*T*
	Mean January temperature (°C)^(3)^	*TJan*
	Mean July temperature (°C)^(3)^	*TJul*
	Mean spring temperature (°C)^(3)^	*TSp*
	Mean summer temperature (°C)^(3)^	*TSu*
	Mean autumn temperature (°C)^(3)^	*TAu*
	Mean winter temperature (°C)^(3)^	*TWi*

Sources: ^(1)^US Geological Survey (1996), ^(2)^Farr and Kobrick (2000), and ^(3)^Agencia Estatal de Meteorología of Spain (AEMET), Ministerio de Medio Ambiente (http://www.aemet.es/es/elclima/cambio_climat/escenarios).

Climate variables were obtained from data supplied by the Agencia Estatal de Meteorología of Spain (AEMET) and digitised using the method explained by Real et al. (2010). Altitude was obtained as a digital coverage by the Land Processes Distributed Active Archive Center, with 100 m spatial resolution. The digital slope was calculated based on altitude using Idrisi (Eastman 2004), according to the methods described in Barbosa et al. (2003). For the topographic variables altitude and slope, we considered the possibility of unimodal responses of the species, by including the quadratic function of these variables (A^2^, S^2^). Exposures to the south (SE) and west (WE) were derived from GlobDEM50 high-resolution digital elevation data, based on raw data from the Shuttle Radar Topography Mission (Farr and Kobrick 2000). For the variable SE, a pixel whose aspect was south was given the value 180, a pixel whose aspect was north was given a value of 0, and pixels with intermediate aspects (westward or eastward) were given intermediate values. The procedure was analogous for the variable WE. Although the resolution scale adopted for all variables was 1 pixel *c*. 1 km^2^, we obtained the values of the variables in the UTM 10 × 10 km squares using the module EXTRACT of Idrisi32 (Clark University, Worcester, MA).

The climatic data resulted from the regional downscaling to Spain of the climate change models produced by the Intergovernmental Panel on Climate Change (IPCC). We used four atmosphere–ocean general circulation models (AOGCMs): CGCM2 from the Canadian Climate Centre for Modeling and Analysis, ECHAM4 from the Max Planck Institut für Meteorologie, and HadAM3H and HadCM2SUL from the Hadley Centre (UK). According to the data obtained from the AEMET, the circulation models CGCM2 and ECHAM4 were run with the conditions forecasted by the Special Report on Emissions Scenarios (SRESs) A2 and B2 (Nakicenovic and Swart 2000), HadAM3H was run with the scenario available A2, and HadCM2SUL was run with the scenario IS92a (Leggett et al. 1992). All the climatic models were run for the periods: 1961–1990 and 2071–2100.

### Model construction

#### Variable selection

We developed distribution models for the species based on topographic variables alone, on climate alone, and on topography and climate together (see Table1). To reduce multicollinearity among variables, we made a preselection of the candidate variables by calculating Spearman correlation coefficients between them, retaining only one out of any set of variables correlated with *r* > 0.8. The relationships between the individual variables and the distribution of the species were tested separately using logistic regression. In this way, we tested a logistic relationship between the species distribution and the variables. This includes a S-shaped relationship (if the logit function covered wide positive and negative ranges), a J-shaped relationship (if the logit function was biased to the negative range), a potential relationship (if the logit was biased to the positive range), and a linear relationship (if the logit function covered narrow positive and negative ranges). We calculated the significance of the score statistic, based on the maximum likelihood estimation (MLEs), and only those variables whose relationship was significant with *α* < 0.05 were retained. To avoid the increase in type I errors arising from the large number of remaining variables (García 2003), we controlled the false discovery rate (FDR) according to Benjamini and Hochberg's (1995) procedure, accepting only those variables that were significant under an FDR of *q* < 0.05. We also used the variance inflation factor (VIF) to quantify collinearity among the remaining explanatory variables in the topographical, climatic, and the topo-climatic models. The square root of the variance inflation factor indicates how many times larger the standard error of a variable is, compared with what it would be if that variable were uncorrelated with the other independent variables in the equation (Zuur et al. 2010).

With the resulting set of variables, we run forward–backward stepwise logistic regression on each subset of predictor variables (topographic, climatic, and topo-climatic). To estimate the relative weight of each variable in the models, we used Wald's test (Wald 1943).

#### The favorability models

On the obtained logistic regression models, we applied the favorability function (Real et al. 2006) and obtained the corresponding favorability (*F*) for the species in each UTM 10 × 10 km cell. This function may be expressed as:

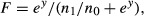
where *F* is the logit link of the favorability function, *e* is the Neperian number, *y* is the logistic regression model equation, and *n*_1_ and *n*_0_ are the numbers of presences and absences, respectively.

Favorability models show how the probability of a species' local presence differs from that expected by chance in the study area, making it easier to differentiate between localities with environmental conditions that are favorable or unfavorable for the species' presence (Acevedo and Real 2012). Unlike other modeling techniques providing probability values, favorability models can discriminate between the effect of environmental conditions and the probability of presence derived from the species prevalence within the study area (Acevedo and Real 2012). Furthermore, favorability models allow for model combinations through fuzzy logic (Acevedo et al. 2010).

#### Model assessment

The Akaike information criterion (AIC) was applied to the obtained models, selecting the most parsimonious (Burnham and Anderson 2002). The goodness of fit of the models was assessed by means of the Hosmer and Lemeshow (2000) test. We assessed the discrimination power of the favorability models by calculating Cohen's kappa coefficient, sensitivity, specificity, and correct classification rate, using the favorability value of *F* = 0.5 as classification threshold (see Fielding and Bell 1997). We also obtained the area under the curve (AUC) to complete the assessment of the discrimination capacity of the different distribution models (see Lobo et al. 2008). Favorability models with higher discrimination capacity were considered to be better models, as all the models were applied to the same dataset.

#### Variation partitioning procedure

As climate and topography are interrelated, we determined the relative contribution of climate in relation to that of topography in the topo-climatic model using a variation partitioning procedure (Muñoz and Real 2006). In this way, we distinguished the apparent effect of climate (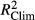), both exclusively and in conjunction with topography, the pure climatic factor (PCF, measured with 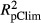), that is, the pure effect of climate on the model variation not affected by topography; the pure topographic factor (PTF, measured with 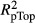), that is, the variation in the model that was due to the pure effect of topography not affected by climate; and the shared climatic factor (SCF, measured with 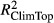), that is, the proportion which was assignable to their shared effect (Legendre 1993; Legendre and Legendre 1998; Randin et al. 2009; see also Muñoz et al. 2013).

### Future projections and fuzzy logic theory

The climatic and topo-climatic favorability models were projected to the future by replacing the values of the different climatic variables in the logit function for the present (*y*_*p*_) by their corresponding values in the period 2071–2100. In this way, we obtained in each cell a value of the logit function for the future (*y*_*f*_) and a corresponding expected future favorability (*F*_*f*_) according to the apparent effect of climate on the species distribution for each AOGCMs -SRESs combination.

Favorability values may be interpreted as the degree of membership of the sites to the fuzzy set of localities favorable to the species (Real et al. 2006, 2010; Acevedo and Real 2012; Muñoz et al. 2013). In this way, we used some fuzzy logic operations (Kuncheva 2001) to calculate, for each future projection, the IOMS features of the forecasted effect of climate change of the species favorability proposed by Real et al. (2010), namely the *increment* in favorability for the species (I), the favorability *overlap* (O), the degree of favorability *maintenance* (M), and the forecasted *shift* in favorability (S) with respect to the 1961–1990 period:

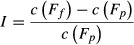


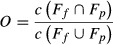


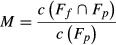


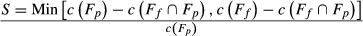


where *c*(*X*) is the cardinality of the *X* fuzzy set, that is, the sum of all cells' membership degrees to the fuzzy set *X*.

1. *F*_*f*_ is the fuzzy set of future favorable areas for the species, and the membership degree of each cell to *F*_*f*_ is defined by the future favorability value for the species in the cell.
2. *F*_*p*_ is the fuzzy set of present favorable areas for the species, and the membership degree of each cell to *F*_*p*_ is defined by the present favorability value for the species in the cell.
3. *F*_*f*_ ∩ *F*_*p*_ is the intersection between future and present favorabilities, and the membership degree of each cell to *F*_*f*_ ∩ *F*_*p*_ is defined by the minimum of the two favorability value for the species in the cell.
4. *F*_*f*_ ∪ *F*_*p*_ is the union between future and present favorabilities, and the membership degree of each cell to *F*_*f*_ ∪ *F*_*p*_ is defined by the maximum of the two favorability values for the species in the cell.

Positive values of increment (I) indicate a favorability expansion for the species, that is, a gain of favorable areas, whereas negative values of I mean a net loss of favorability areas for the species. High values of overlap (O) indicate that the distribution of the future local favorability values is predicted to be similar to that shown at present. Maintenance (M) indicates the degree to which current local favorability values are predicted to persist in the future, so that low values of M are of more conservation concern that high M values. Favorability shift (S) measures the proportion of the present favorability that is predicted to be lost in the future but may be compensated with new favorability opportunities elsewhere.

Applying the expression *y*_*f*1_ = *y*_*p*_ + *ρ*(*y*_*f*_ − *y*_*p*_), we calculated the minimum (if the apparent effect is inflated) or the maximum (if the apparent effect is obscured) climatic effect over the species distribution, where *y*_i_ represents the logit functions of the corresponding probability models, and *ρ *= 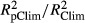. We applied the favorability function (Real et al. 2006) to obtain the expected future favorability according to the pure effect of climate (*F*_*f1*_), so that *F*_*f*_ and *F*_*f*1_ represent the limits of the forecasted effects of climate change on the spatial distribution of the favorability for the species.

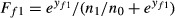


### Considering behavioral plasticity

By modeling the distribution of the species with the variables related to topography, we obtained the potential range of the eagle only considering nesting substrate availability, according to Bonelli's eagle current use of breeding habitat; by modeling the species distribution using only climatic variables, we established the current climatic favorability for the species; and by computing the fuzzy intersection between the topographic and the climatic models, we identified those areas that were simultaneously favorable according to climate and nesting substrate availability. In this way, we (1) discarded areas with availability of cliffs to breed that were climatically unfavorable, and (2) discarded climatically favorable areas where cliff availability was low.

By modeling the distribution considering both topographic and climatic variables together, we identified those areas that were favorable for the species due to a combination of climate and topography, allowing a certain compensation of favorability between the two factors, which implies a certain plasticity in the nesting behavior of the species in those climatically favorable areas, which is the characteristics we are aiming to include in the models. We projected the models representing the maximum climatic effect over the species distribution, as they represent the maximum of opportunity to take advantage of by the species through behavioral evolution or plasticity.

We assessed the models' performance both outside and inside the Spanish Mediterranean area (Andalusia, Murcia, Valencia, and Catalonia; see Fig.1), as the latter is the climatically favorable region for the species, and where this kind of evolutionary or phenotypically plastic compensation could putatively be currently occurring.

The extrapolation of the topo-climatic models to the future yielded future favorabilities for the species on the assumption that a lack of topographic favorability can be compensated by an increase in climatic favorability. This will be true only if an increase in climatic favorability is forecasted and the species may change its nesting behavior, and so those forecasts include the realization of the behavioral plasticity of the species into the predictions. However, the species could remain constrained by the current plasticity in nesting behavior in Spain, which was included in the current topo-climatic model, only being able to increase its nesting distribution in new climatic favorable areas that are also topographically favorable. This was evaluated using fuzzy logic operations to obtain, for each climate change scenario predicting an increase in climatic favorability, the following model:

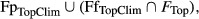
where Fp_TopClim_ is the current topo-climatic favorability, Ff_TopClim_ represents the forecasted maximum topo-climatic favorability, and F_Top_ is the topographic favorability. This model will forecast future favorability areas on the assumption that the species will not increase its current plasticity in nesting behavior. In this way, Ff_TopClim_ represents favorability forecasting in case of complete realization of the evolutionary capacity of the species, whereas Fp_TopClim_ ∪ (Ff_TopClim_ ∩ F_Top_) represents the favorability forecasting in a scenario of no behavioral change.

## Results

### The favorability models and fuzzy logic theory

**Figure 4:**
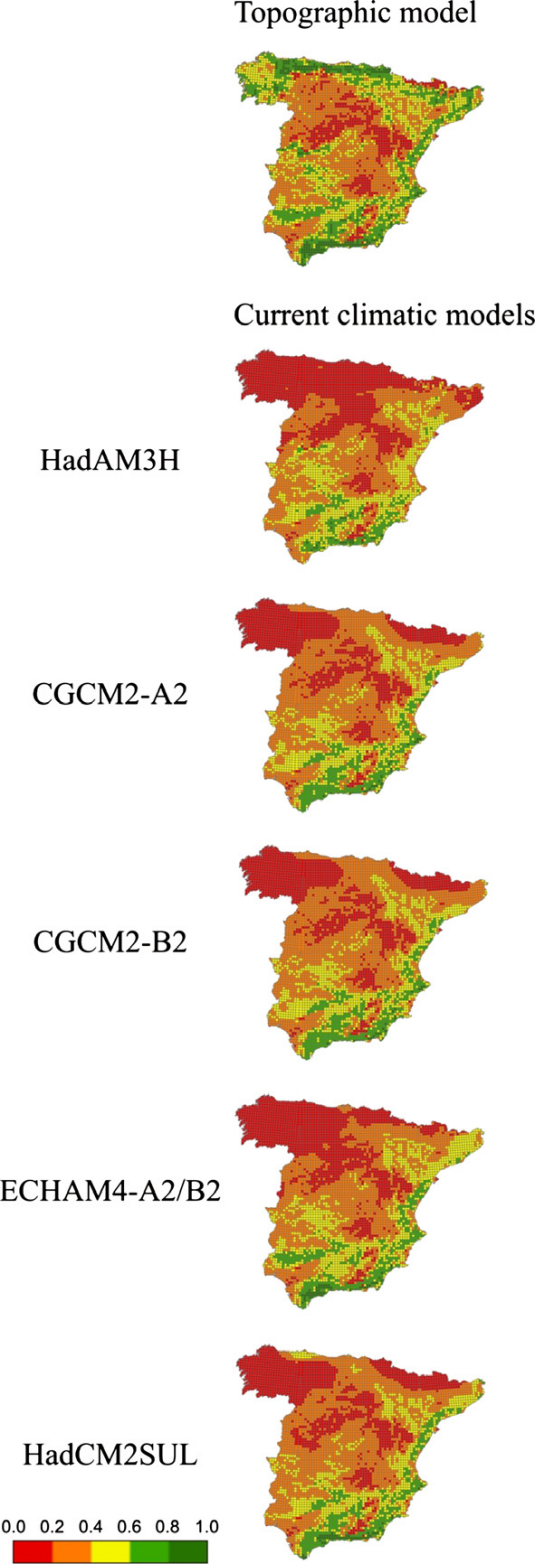
Current topographic and climatic favorabilities for *A. fasciata* in Spain. Climatic favorabilities are calculated for each AOGCM-SRES combination.

We obtained significant models for the current favorability according to both the topographic and the climatic factors, and for the two factors combined. The models based on only topography and only climate produced very different favorability outputs (Fig.4), but their overall explanatory and discrimination capacity, and classification performance were similar (Table2). The topographic model predicts favorable areas in most of the Spanish mountain ranges, even in the north, where the species is absent, while the climatic models forecast as favorable mostly the Mediterranean region in all combination of AOGCM and SRES, which indicate that the true role of topography is only apparent after considering climate.

**Table 2:** Models obtained for each explanatory factor and values obtained to assess them. Variables codes as in Table1

Factor	AOGCM-SRES	Variables	AIC	Kappa	Sens.	Spec.	CCR	AUC	H-L
Topographic		*A, A*^*2*^*, S, S*^*2*^*, OrS, OrW*	3432.81	0.233	0.703	0.705	0.705	0.766	49.384*
Climatic	HadAM3H	*PWi, Tsp, TSu,TWi*	3410.30	0.199	0.543	0.760	0.733	0.758	35.338*
	CGCM2-A2	*PSp, PAu, TAu*	3417.97	0.191	0.732	0.640	0.652	0.762	67.640*
	CGCM2-B2	*PSp, PAu, TAu*	3416.58	0.192	0.732	0.641	0.653	0.762	67.545*
	ECHAM4-A2/B2	*PSp, PSu, PAu, PWi, TSp, TSu, TAu*	3395.91	0.199	0.726	0.654	0.663	0.758	36.906*
	HadCM2SUL	*PSp, PSu, PAu, PWi, TSp, TAu*	3375.42	0.207	0.748	0.650	0.663	0.768	15.406*
Topo-climatic	HadAM3H	*A, A*^*2*^*, S, S*^*2*^*, PSp, PWi, TAu*	2856.91	0.363	0.773	0.785	0.783	0.855	6.734 n.s.
	CGCM2-A2	*A*^*2*^*, S, S*^*2*^*, PSu, PAu, PWi, TSp, TSu, TAu*	2864.67	0.351	0.774	0.776	0.775	0.858	12.975 n.s.
	CGCM2-B2	*A, A*^*2*^*, S, S*^*2*^*, PSu, PAu, PWi, TSu*	2865.11	0.352	0.776	0.776	0.776	0.857	7.533 n.s.
	ECHAM4-A2/B2	*A, A*^*2*^*, S, S*^*2*^*, OrW, PAu, TSu*	2878.27	0.347	0.770	0.775	0.775	0.854	11.995 n.s.
	HadCM2SUL	*A, A*^*2*^*, S, S*^*2*^*, PSu, PAu, PWi, TSp, TSu*	2875.36	0.353	0.767	0.780	0.778	0.852	8.944 n.s.

Parsimony was assessed using Akaike information criterion (AIC), and goodness of fit was assessed with the Hosmer and Lemeshow test (H-L).

* : *P* < 0.01 and n.s.: *P* > 0.01. Cohen's kappa, sensitivity (Sens.), specificity (Spec.), and correct classification rate (CCR) have been calculated using the favorability value of *F* = 0.5 as the classification threshold and area under the receiver operating characteristic curve (AUC).

Variables related to aspect were included in the topographic model (Table2), but in the topo-climatic model, they tended to be excluded. Both precipitation and temperature variables were included in climatic models. Scenarios A2 and B2 applied to the circulation model ECHAM4 produced the same precipitation and temperature values, and thus the same models, whereas they differed in the model CGCM2. This is why we named these climatic and topo-climatic models ECHAM4-A2/B2, CGCM2-A2 and CGCM2-B2, respectively (see Table2).

The combined topo-climatic models showed the best explanatory capacity according to the AIC values, as well as the best discrimination capacity according to AUC, and classification performance according to Kappa, sensitivity, specificity, and CCR. They were also the best calibrated models according to the Hosmer and Lemeshow goodness-of-fit test, being the only models showing no significant differences between the observed and the expected proportions of presences in the different classes of probability (Table2).

**Figure 5:**
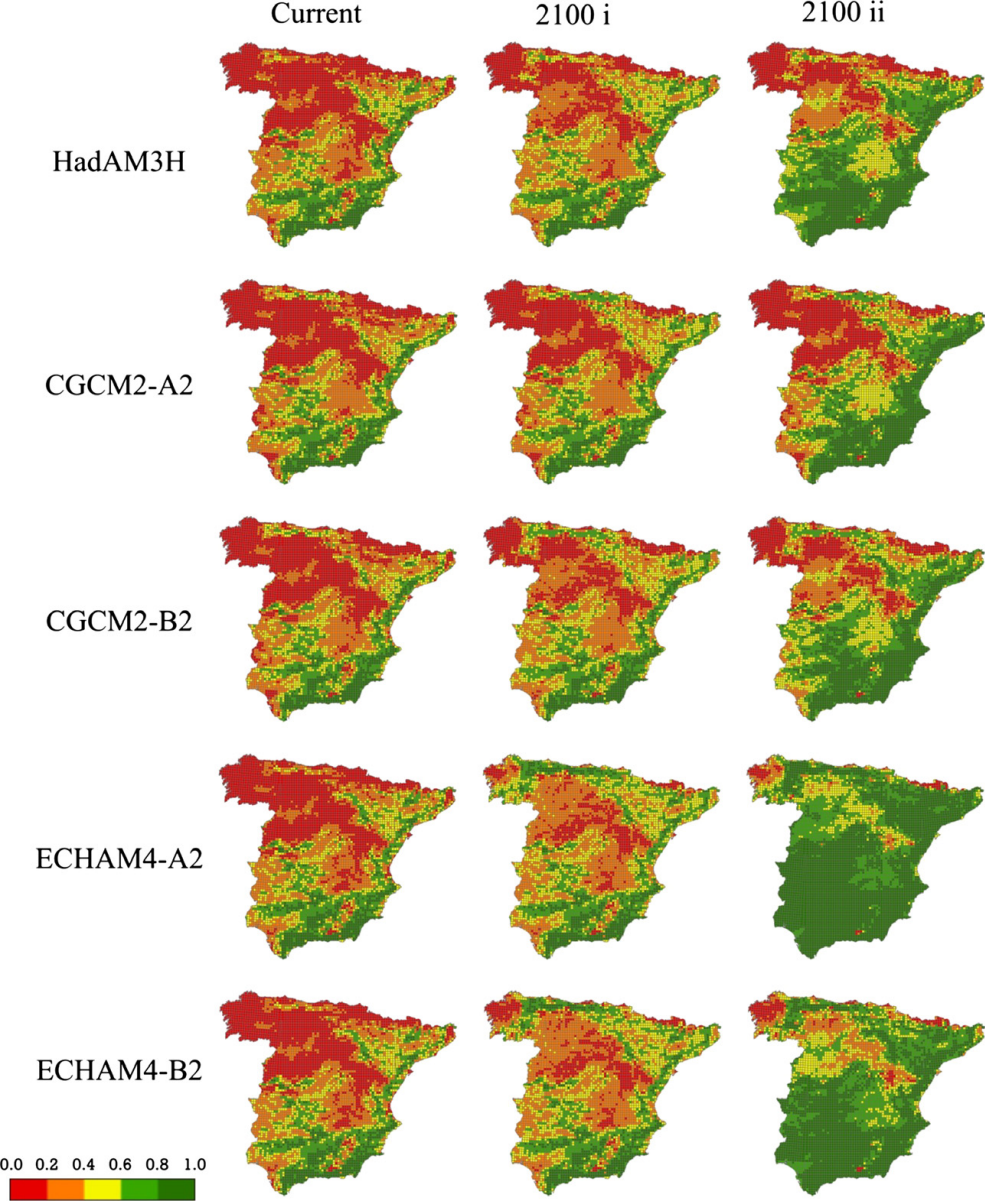
Favorability forecasted at each 10 km × 10 km UTM square of mainland Spain for *A. fasciata* according to each topo-climatic model and for each period. i: assuming maintenance of current breeding habits, restricted to mountainous areas and ii: considering the capacity to evolve by breeding outside mountainous regions in new climatically favourable areas.

All the models included variables with a VIF lower than 10, except those corresponding to the circulation model CGCM2, which nevertheless do not seem to produce inflated projections according to Fig.5. The pure effect of climate on the topo-climatic models of the species' distribution was always more important than the pure effect of topography (Table3). The shared effect of climate and topography was always negative which indicates that the effect of one factor is obscured by the other in the amount expressed by the negative shared effect shown in Table3. The *ρ* value was always higher than 1 what indicates that the pure climatic factor is greater than the apparent (see Table3).

**Table 3:** Results of the variation partitioning of the topo-climatic favorability model for each combination of AOGCM and SRES. Values shown are the percentages of variation explained exclusively by the pure topographic factor (PTF), by the pure climatic factor (PCF), and by their shared effect (SCF). The *p* value indicates the proportion of pure climatic factor in relation to whole-climatic factor

Model	HadAM3H	CGCM2-A2	CGCM2-B2	ECHAM4-A2/B2	HadCM2SUL
PTF	39.8	41.8	48.2	45.3	37.3
PCF	73.1	70.2	61.6	71.7	70.9
SCF	−12.9	−12.0	−9.8	−17.0	−8.2
*ρ*	1.214	1.206	1.189	1.311	1.131

The fuzzy intersection between the topographic and climatic models had an overall discrimination capacity similar to that of the topo-climatic model according to AUC (0.836–0.855), better classification performance according to Kappa (0.366–0.430), CCR (0.845–0.860), and specificity (0.883–0.919), and worse sensitivity (0.448–0.579) that those of the topo-climatic models.

The topographic model discriminated better in the Mediterranean Spanish region than in the rest of Spain, whereas the opposite was true for the climatic model (Table4). The topo-climatic model discriminated similarly in both regions and was the best discriminatory model in both areas, whereas the fuzzy intersection between the topographic and climatic models discriminated worse in the Mediterranean region than in the rest of Spain.

**Table 4:** Discrimination capacity for each model in Spain, the Spanish Mediterranean region (consisting of Andalusia, Murcia, Valencia, and Catalonia), and outside the Spanish Mediterranean region. Values of AUC are shown

Factor	AOGCM-SRES	All of Spain	Only Medit. Spanish region	Only non-Medit. Spanish region
Topographic		0.766	0.823	0.692
Climatic	HadAM3H	0.758	0.669	0.740
	CGCM2-A2	0.762	0.567	0.764
	CGCM2-B2	0.762	0.570	0.765
	ECHAM4-A2/B2	0.758	0.617	0.752
	HadCM2SUL	0.768	0.610	0.755
Topo-climatic	HadAM3H	0.855	0.837	0.833
	CGCM2-A2	0.858	0.829	0.840
	CGCM2-B2	0.857	0.831	0.837
	ECHAM4-A2/B2	0.854	0.827	0.838
	HadCM2SUL	0.852	0.835	0.830
Topog. ∩ Climat.	HadAM3H	0.836	0.787	0.821
	CGCM2-A2	0.855	0.829	0.844
	CGCM2-B2	0.855	0.829	0.845
	ECHAM4-A2/B2	0.845	0.815	0.831
	HadCM2SUL	0.849	0.829	0.824

### The future projections and the behavioral plasticity

All AOGCMs-SRESs models forecasted an increase in climatic favorability for the species, except HadCM2SUL which yielded a favorability increment of I = −0.4366 and was not analyzed for a change in nesting behavior using fuzzy logic. All models, regardless of whether they assumed a further change in nesting habits, predicted maintenance of the current favorability for Bonelli's eagle, but those assuming plasticity in nesting behavior predicted notably larger increments in favorability (Table5).

**Table 5:** Values of the rates of increment (I), overlap (O), maintenance (M), and shifting (S) of favorability forecasted for 2100 with respect to the 1961–1990 period

AOGCM-SRES	I	O	M	S
Maintaining the current breeding habits				
HadAM3H	0.1235	0.8900	1.0000	0.0000
CGCM2-A2	0.0968	0.9117	1.0000	0.0000
CGCM2-B2	0.1592	0.8627	1.0000	0.0000
ECHAM4-A2	0.2795	0.7816	1.0000	0.0000
ECHAM4-B2	0.2523	0.7985	1.0000	0.0000
Assuming plasticity				
HadAM3H	0.5175	0.6590	1.0000	0.0000
CGCM2-A2	0.3410	0.7426	0.9976	0.0024
CGCM2-B2	0.4800	0.6756	0.9999	0.0001
ECHAM4-A2	1.1711	0.4606	1.0000	0.0000
ECHAM4-B2	0.8987	0.5267	1.0000	0.0000

The present favorability for Bonelli's eagle and the projected future favorabilities according to the climatic conditions forecasted for the year 2100 by each AOCGM and SRES combination are shown in Fig.5, assuming (1) maintenance of current breeding habits mostly restricted to mountainous areas, which presumes that the species will only colonize those mountains becoming climatically favorable or (2) capacity to evolve by breeding outside mountainous regions in emerging climatically favorable areas.

## Discussion

### Considering behavioral plasticity in species distribution models

This study demonstrates that projected species-range changes expected to occur under climate change could be wrongly estimated if the capacity of species to adapt to new conditions is completely ignored. Thus, it is likely the number of species committed to future extinction or to become severely threatened as a consequence of climate change, in the coming years, may be not as high as recent studies suggest (Thomas et al. 2004; Thuiller et al. 2005; Sinervo et al. 2010; Barnosky et al. 2011; He and Hubbell 2011; Moritz and Agudo 2013).

Environmental changes have been shown to shift the average phenotype of well-adapted populations further from its optimal value, and thereby to reduce mean fitness (Bell and Collins 2008). Species may adjust to these changes through phenotypic plasticity, although if offspring encounter different conditions from their parents and changes are too great to reproduce successfully or survive, the population may decline (Bell and Collins 2008). But if the environmental change goes in the same direction as the vital needs of the species, the environmental favorability may improve and, hence, favor population increase and dispersion. If projections of climate change, both of general circulation models and greenhouse gasses are consistent, and the lack of topographical favorability is compensated by an increase in climatic favorability, an increase in the distribution of this endangered species is expected. This would entail a behavioral change in the species, that is, an alteration in nesting requirements, including not only mountains that are currently unfavorable and will become favorable but also lowlands, which are at present rarely occupied in the studied area.

A general future increase in the environmental favorability for Bonelli's eagle in mainland Spain was predicted when using most combinations of AOGCMs and SRESs. Although this result concerns only one species, and thus, is not enough to warrant any generalization, it agrees with those of Real et al. (2010) for another mountain vertebrate species, the Iberian wild goat *Capra pyrenaica*, and with those of Aragón et al. (2010), who predicted an increase in climatic favorability for some Iberian vertebrates. The detailed analysis, in which factors other than climate are considered when assessing the effects of climate change, may provide cause for optimism in conservation, although there are species for which adverse effects are predicted (Walther et al. 2002; Araújo et al. 2011). An increase in favorability in areas currently unfavorable may result in an increase in the distribution of the species, but extrinsic factors, such as biotic and anthropogenic interactions, and factors intrinsic to the species, may interfere in this process. Our models pinpoint areas of emergent favorableness that greatly differ in favorability if the possibility of the species to change their breeding habits is considered or not.

At present, we find different nesting habits of Bonelli's eagle in relatively close areas. In southern Portugal, 94% of breeding pairs are tree-nesters (Palma 2010), while in southern Spain, this proportion is reversed, with more than 95% of pairs breeding on cliffs. It is worth noting that in both regions, both cliffs and forested areas can be found. If climate change leads to increased climatic favorability for the species in the near future, it would be reasonable to consider the possibility of tree-nesting habits to increase in Spain. The behavioral diversity for this ecologically relevant trait already exists, as the necessary requirement for the adaptive behavioral changes to occur.

Predicted favorability models offered in this study show two extremes: one considering that the species will still be linked to mountainous areas, and another assuming that the species will take full advantage of the possibility to breed on trees in Spain, as it already does in Portugal. Likely, the species will follow a course between these extremes, and only future monitoring of the population will help to determine the extent to which behavioral change occurs. In this regard, it would be interesting to incorporate demography and dispersal into the model to obtain more robust predictions to guide conservation planning at both local and global scales (Moritz and Agudo 2013).

### The modeling approach

Predicting the future distribution of a species from a part of its range, as we did, could be oblivious to the variation in climate tolerance that is not present in the studied area (Thuiller et al. 2004; Hattab et al. 2014). However, in a global model, the relationship between climate and nonclimatic factors, and their separate and combined effects on the species distribution, is averaged throughout the species range (in this case from Portugal to China, including northern Africa and Indonesia), while some factors may be more (or less) critical than average in specific zones of the species range. More importantly, our aim with this manuscript was to consider the possibility of including behavioral plasticity and evolutionary scenarios in SDM, as a more realistic approach to the assessment of the consequences of global change on species' distribution. These evolutionary scenarios are driven by more local characteristics and cannot be averaged over a global scale, which makes the possibility of using a global model less preferable in our approach. A global model updated with Spanish data would take advantage of both pros (global and regional) and could be preferable, but the distribution of the species is not well known at global scale with necessary detail, and thus, this option is not currently available.

Species distribution models need to formulate the relationship between distributions and environmental variables explicitly to become appropriate tools to generate hypotheses about how species respond to spatial and environmental variability and to provide insights into the potential response of species to regional climate change (Moritz et al. 2008; Márquez et al. 2011; Triviño et al. 2011). Our methodological approach to obtain models for each explanatory factor guarantees that every variable included in the model is significantly related to the species distribution independently of the effect of other variables in the model. The use of a stepwise procedure for combining different influential factors produced better results according to AIC and AUC values than the use of the explanatory factors considered independently and provided a valuable approach to integrate different potential drivers of changes in species' distribution in the framework of climate change scenarios.

The entry order of the variables in the stepwise procedure provides additional valuable information, as this order largely depends on the scale on which the variables operate, the first variables explaining broad-scale distribution of the species and the last acting on finer-scale (local) distribution patterns (see Fig.3 in Muñoz et al. 2005). In the case of the topographic model, those variables related to aspect entered at the end, which suggests that these variables are decisive only at a more local level. In the topo-climatic models, aspect tended to be excluded, suggesting that it may be a surrogate of (micro) climate, and so it may be redundant in a topo-climatic model at this scale. This information on the scale at which each factor is relevant is not retained in the final function, as the values of the parameters associated with each variable are reassessed according to the collinearity with the other variables included in the model.

### The true role of topography and climate

Scale could also be considered relevant in the action of climate and topography. Hattab et al. (2014), for instance, applied the concept of “hierarchical filters” to combine the predictions of a climatic and a topographic model developed for two different spatial extents (global and local), on the assumption than climate operate at a larger spatial scale. In our case, the performance of the topographic and climatic models was very similar (Table2), which seems to indicate that both factors are operating at the same scale within Spain.

The combination of factors produced better models than the consideration of climate alone, which suggests that topographic variables play an important role in determining mountain species distribution, and its dynamics over time. Davis et al. (1998) and Hattab et al. (2014), for example, questioned the validity of climatic models for forecasting future species distributions arguing that many factors other than climate influence species' ranges. Because of this, we considered more appropriate to forecast future changes in environmental favorability taking into account the relevant factors determining the species' distributions instead of building new models that are based on climate change variables only. We recommend assessing the additive importance of different potentially influential factors on the distribution of species before addressing the task of forecasting future situations in climate change scenarios. It is important to remark that in the combined models, the driving effect of a changing climate may be compensated by the inertia caused by more constant influential factors, such as topography in our case.

Although the climatic and topographic models were very similar in the values obtained in the assessing measures shown in Table2, the pure effect of climate on the combined models was more important than the pure effect of topography in all combined models (Table3). If climate affects species' distribution more than expected according to the mere comparison of the climatic and topographic models, species could be more responsive to a change in climate, including a behavioral change, than what intuition suggests. In our case, we could consider the possibility that this eagle tended to occupy Mediterranean mountains in Spain not mainly due to the topography but mostly because these areas are climatically favorable, and mountains happen to be prevalent in these areas. This could explain why the percentage of breeding pairs nesting on trees is so high in southern Portugal, compared with Spain. In Portugal, Bonelli's eagle would also occupy areas climatically favorable, but in this case, these areas are not so mountainous, and therefore, the species tends to nest on trees. The higher discrimination capacity of the climatic model outside the Spanish Mediterranean region compared with the topographic model also suggests that it is the effect of climate, more than that of topography, which is critical in restricting the distribution of the species there.

This approach could be used as such for mountain species in general, which may be of particular interest given that mountain ecosystems seem to be particularly sensitive to global warming worldwide (Wilson et al. 2005; Pauli et al. 2007). Wilson et al. (2007) and Gasner et al. (2010) pointed out that mountain species ranges might respond more rapidly to climate change because mountains often retain more intact habitats than lowland landscapes, and because these species can track climate change over shorter distances. Taking into account, the topography might produce more realistic predictions in climate change scenarios.

### Implications for future research

Currently, it is a challenge to include the behavioral and physiological plasticity of species when modeling species distribution and progress is needed in this direction (Harvey et al. 2012; Pearson et al. 2014), especially when considering the potential consequences of global change on endangered species. Being able to accurately determine the potential adaptive response of species would allow us to better predict their future situation and to optimally manage and protect wildlife resources (Rapacciuolo et al. 2014). Ignoring phenotypical plasticity when projecting to the future, the changes in environmental drivers normally under- or overestimate the predicted consequences and effects on species distributions. Our results indicate that it is possible to consider different adaptive responses of species when modeling the effects of climate change on species distribution. Using this approach when possible, we could strengthen the science behind conservation planning in the present situation of rapid climate change.

Species distribution modelers rarely include phenotypical traits when characterizing species distributions and evolutionary biologist rarely use the extensive data available about spatial environmental variation and climate change. There is considerably more to be learned by applying new methods that appropriately take into account the potential of evolutionary processes. This is especially of interest given that adaptive responses do not need a long-time scale to occur (Thompson 1998; Van Doorslaer et al. 2007; Sultan et al. 2012). Contemporary evolution is probably more important than customarily assumed and is likely to mediate the response of species, populations, communities, and ecosystems to both gradual and rapid environmental changes (Mergeay and Santamaría 2012). To our knowledge, this is the first study including both the appearance of phenotypical adaptations and the lack of behavioral changes in modeling the effects of climate change on species distribution, producing local predictions of biogeographic change.

Many species have some phenotypic or behavioral plasticity and evolutionary potential, and our approach allows the inclusion of evolutionary scenarios into the model projections to the future. In this respect, the approach may be generalized for other species affected by other factors. Validation of this kind of results will require population and genetic monitoring, and focused empirical studies to assess and better understand the impact of climate change.
